# The Human Blood Fluke, *Schistosoma mansoni*, Harbors Bacteria Throughout the Parasite's Life Cycle

**DOI:** 10.1093/infdis/jiad288

**Published:** 2023-07-24

**Authors:** Fabio Formenti, Alba Cortés, Michela Deiana, Susannah Salter, Julian Parkhill, Matt Berriman, Gabriel Rinaldi, Cinzia Cantacessi

**Affiliations:** Department of Veterinary Medicine, University of Cambridge, Cambridge, United Kingdom; IRCCS Sacro Cuore Don Calabria Hospital, Verona, Italy; Department of Veterinary Medicine, University of Cambridge, Cambridge, United Kingdom; Departament de Farmàcia, Tecnologia Farmacèutica I Parasitologia, Facultat de Farmàcia, Universitat de València, Burjassot, Spain; IRCCS Sacro Cuore Don Calabria Hospital, Verona, Italy; Department of Veterinary Medicine, University of Cambridge, Cambridge, United Kingdom; Department of Veterinary Medicine, University of Cambridge, Cambridge, United Kingdom; Wellcome Trust Sanger Institute, Hinxton, United Kingdom; Wellcome Centre for Integrative Parasitology, Institute of Infection, Immunity and Inflammation, College of Medical, Veterinary and Life Sciences, University of Glasgow, Glasgow, United Kingdom; Wellcome Trust Sanger Institute, Hinxton, United Kingdom; Department of Life Sciences, Aberystwyth University, Aberystwyth, United Kingdom; Department of Veterinary Medicine, University of Cambridge, Cambridge, United Kingdom

**Keywords:** schistosomes, fluorescence in situ hybridization, parasite-associated microbiota

## Abstract

While symbiotic relationships between invertebrates and bacteria have been extensively described, studies of microbial communities inhabiting parasitic worms remain scarce. Exploring the microbiota associated with helminths responsible for major infectious diseases will inform on parasite biology, host-pathogen interactions, and disease pathophysiology. We investigated the presence of microorganisms inhabiting tissues of the human parasite *Schistosoma mansoni*. In situ hybridization using a pan-bacterial 16S rRNA gene probe revealed bacteria colonizing key developmental stages that were successfully removed after antibiotic treatment of live parasites. Understanding the composition and function of the *S. mansoni*-associated microbiota may lead to the development of novel microbiome-targeting control strategies

## Teaser

In this study, we provide experimental evidence that *Schistosoma mansoni*, a species of blood fluke infecting millions of people worldwide, harbors a microbiota that may be targeted in future studies aimed to develop novel strategies for parasite control.

Schistosomiasis is a neglected tropical disease affecting >250 million people worldwide [1], caused by *Schistosoma* blood flukes. The parasite life cycle involves both vertebrate, including human, and invertebrate hosts (ie, freshwater snails) [1]. Humans become infected when free-living larvae (cercariae), released in freshwater from *Biomphalaria* snails, penetrate the skin and become schistosomula. Schistosomula migrate within the circulatory system via the lungs to the liver vasculature, where they develop and pair up before migrating to the mesenteric veins (in the case of *Schistosoma mansoni*). Four weeks post-infection, female worms begin to lay eggs that traverse the lining of the mesenteric vessels, migrate through the intestinal wall to the lumen and are excreted with host feces. In fresh water, the eggs hatch ciliated miracidia that infect the snail, undergo asexual multiplication, and develop from primary and secondary sporocysts to cercariae. The pathophysiology of schistosomiasis is mainly driven by parasite eggs becoming trapped in the liver and intestines, promoting inflammation, granuloma formation, and fibrosis [1]. Control of schistosomiasis in endemic areas has long relied on the administration of a single drug, that is praziquantel, delivered in mass drug administration programs [1]. However, praziquantel is active only against adult worms, reinfections are common, and tissue lesions persist after treatment. The reliance on a single drug is likely to lead to the emergence of drug-resistant parasites [1]; thus, new drugs and eventually vaccines are desperately needed.

Fundamental knowledge of schistosome biology and interactions with their hosts may reveal exploitable vulnerabilities that could be targeted in the development of novel and sustainable control strategies. One emerging area of interest focuses on the occurrence of communities of microorganisms that inhabit the parasitic worm, collectively known as the worm-associated microbiota [eg,. 2]. Microorganisms closely affiliated with parasitic worms have been described in filarial nematodes and identified in the reproductive tissues of some digenean trematodes [eg, 2]. To date, a single study demonstrated that the tegument and gastrodermis of *Schistosoma japonicum* adult worms are inhabited by populations of bacteria whose functions remain unknown [3]. While this preliminary evidence is promising, determining whether schistosome-associated bacteria represent a potential target for intervention relies on the acquisition of additional knowledge of their location and propagation throughout the parasite life cycle. In this study, we applied fluorescence in situ hybridization (FISH) to explore the occurrence of *S. mansoni*-associated bacteria throughout key developmental stages. We detected a strong 16S rRNA signal associated to the apical/lateral glands of eggs and miracidia, to the acetabular glands and oral/ventral suckers of cercariae, and to the gut and tegument of adult worms. Our findings indicate that propagation of bacteria associated with *S. mansoni* may occur vertically via the eggs.

## METHODS

The life cycle of *S. mansoni* (NMRI strain) was maintained at the Wellcome Sanger Institute. The Animal Welfare and Ethical Review Body of the Wellcome Sanger Institute approved all regulated animal procedures conducted under the Home Office Project License P77E8A062.

*S. mansoni* developmental stages were obtained from experimentally infected outbred TO mice as follows: adult worms were collected by portal perfusion; eggs were isolated by collagenase-digestion of infected livers; and miracidia hatched from eggs incubated in water under light [4]. Cercariae were recovered from experimentally infected *Biomphalaria glabrata* [4]. Specimens were fixed in 4% paraformaldehyde in 1× phosphate-buffered saline-0.3% Triton (PBS-T) overnight at 4°C, dehydrated in increasing concentrations of methanol in 1× PBS-T and stored in 100% methanol at −20°C until use. Fixed specimens were rehydrated, through a decreasing methanol concentration series, and paraffin embedded. For each specimen, four 4-µm thick sections were obtained; 1 section was stained with hematoxylin/eosin to assess parasite morphology, while the remaining 3 sections were subjected to FISH, using the KBI-60007 Tissue Digestion Kit I (Leica Biosystems) according to manufacturer's instructions. Briefly, sections were incubated for 1 hour at 70°C and deparaffinized in xylene for 10 minutes. Specimens were rehydrated in 100%, 85%, and 70% ethanol, for 3 minutes each, before washing in water for 1 minute at room temperature (RT). Sections were placed in 0.01 M sodium citrate at 96°C for 15 minutes and incubated for 5 minutes at RT in approximately 200 µL of pepsin solution. Thereafter, specimens were washed in water for 1 minute and in 2× saline-sodium citrate (SSC) buffer for 5 minutes before dehydration with 1-minute washes in 70%, 85%, and 100% ethanol. Sections were incubated with 10 µL of a 100 µM fluorescent probe (universal bacterial probe or control nonsense probe) for 2 hours at 46°C in total darkness. An additional section, incubated with water under the same conditions, was included as control. The Eubacteria probe EUB338 (Eurofins Genomics) was used that includes part of the 16S rRNA gene, double-labelled with ATTO 594 (5′-ATTO594-GCTGCCTCCCGTAGGAGT-3′) [5]. The nonsense probe, Non-EUB338 (Eurofins Genomics), with a complementary sequence to the EUB338 probe and the same fluorophore (ATTO-594) was used as a negative control for non-specific binding [5]. Following probe incubation, sections were washed in 200 µL of 2× SCC/0.3% Igepal lysis buffer for 2 minutes at RT, followed by a wash with 0.4× SCC/0.3% Igepal lysis buffer for 2 minutes at 72°C. Samples were treated with 0.1% Sudan Black B (w/v) (Sigma-Aldrich) for 20 minutes, to reduce autofluorescence , then dehydrated with 1-minute washes in 70%, 85%, and 100% ethanol, before mounting in mounting medium with DAPI (4′, 6-diamidino-2-phenylindole). Fluorescence was evaluated using a Leica SP5 AOBS confocal microscope, with a 40× oil immersion HC PL APO CS2-NA 1.3 objective.

To evaluate the susceptibility of *S. mansoni*-associated bacteria to antibiotics, freshly collected mixed-sex adult worms were cultured at 37°C, 5% CO_2_ in Dulbecco’s Modified Eagle’s Medium (DMEM) complemented with 10% fetal bovine serum, in the presence or absence of a wide-spectrum antibiotic cocktail consisting of 1000 U/mL penicillin, 1000 μg/mL streptomycin, and 100 μg/mL kanamycin (final concentrations). Culture media and antibiotic cocktail were replaced daily for 3 days. Aliquots of approximately 5 worm pairs from the antibiotic-treated or control group were collected daily for 3 days and processed for FISH.

## RESULTS AND DISCUSSION

**Figure 1.**
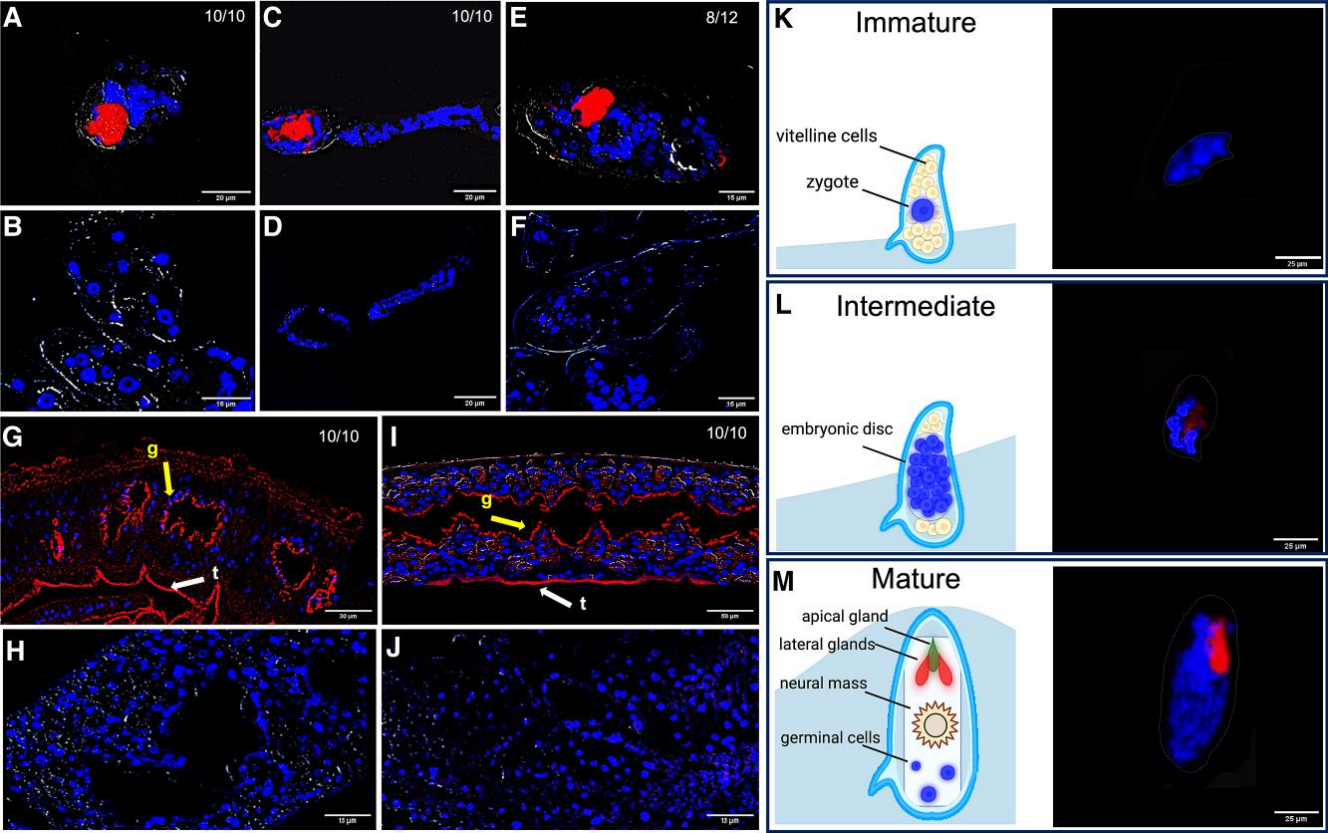
Fluorescence in situ hybridization reveals bacteria in *Schistosoma mansoni*. Bacteria-specific fluorescent signal (red) in representative specimens of *S. mansoni* developmental stages and corresponding negative (ie, irrelevant probe) controls: miracidia (*A* and *B*, n = 10); cercariae (*C* and *D*, n = 10); eggs (*E* and *F*, n = 12 mature eggs); male adult worms (*G* and *H*, n = 10); and female adult worms (*I* and *J*, n = 10). Blue, DAPI-stained nuclei. Yellow arrow, g, gut; white arrow, t, tegument. For each developmental stage, the number of parasites returning a positive fluorescent signal, out of a total number of screened specimens, is indicated. *K–M*, Diagrams (left) and representative images (right) of immature (*K*), intermediate (*L*), and mature (*M*) eggs, respectively. Blue, DAPI-stained nuclei. For statistical analyses, eggs were classified as immature (Jurberg scores 0–5) or mature (Jurberg scores 6, 7) [6]. The statistical significance of the association between egg development and detection of 16S rRNA signal was tested by Fisher exact test performed on a group of 62 randomly selected eggs (n = 62; *P* value = .00029). Scale bars as indicated.

To explore the occurrence of *Schistosoma*-associated bacteria, we employed FISH using a pan-bacterial 16S rRNA gene-specific probe that revealed a strong signal in miracidia, cercariae, eggs, and adult worms. Careful microscopical examination of the specimens returning positive 16S rRNA signals did not reveal any structural damage that may have led to bacterial contamination, thus providing confidence in the robustness of our data. In the miracidium, the signal was localized to the apical and lateral glands (Figure 1*A* and 1*B*, and Supplementary Figure 1). These glands contain droplets and electrodense granular material secreted during invasion of the snail tissue [7]. These secretions consist of proteases, including acidic endopeptidases, and other excretory-secretory products involved in tissue invasion and immune modulation [8]. Cercariae also returned a strong 16S rRNA signal in correspondence to the pre- and postacetabular glands (Figure 1*C* and 1*D*, and Supplementary Figure 1). These glands open to the anterior tip of the cercaria head through ducts packed with secretory bodies that contain proteases involved in skin degradation, cercaria-schistosomulum transformation, and immune-evasion mechanisms [9]. No molecules have thus far been identified in the secretory glands of miracidia or cercariae that may originate from bacteria colonizing these organs. The origin, composition, and functional roles of bacteria (or their metabolites) inhabiting the secretory glands of schistosome larvae during host penetration and infection establishment remain unknown. Eggs of *S. mansoni* were classified according to their embryonic development [9]. A strong 16S rRNA signal was detected within the mature embryo, with a fluorescence pattern and location similar to the one identified in miracidia (Figure 1*E*, 1*F*, and 1*M*, and Supplementary Figure 1), while no 16S rRNA signal could be detected in immature eggs and underdeveloped embryos (Figure 1*K* and 1*L*) (Fisher exact test; n = 62; *P* = .00029; details provided in legend to Figure 1).

To the best of our knowledge, our study is the first to describe the occurrence of bacteria in mature eggs, miracidia, and cercariae of schistosomes. Distantly related trematodes are known to be permanently colonized by obligate intracellular *Neorickettsia* bacteria [2], that are occasionally transferred to vertebrate host tissues over the course of infection, causing severe diseases [10]. *Neorickettsia* spp. have been localized to different tissues of adult worms, including the vitelline cells of the liver fluke *Fasciola hepatica* [10]. This localization suggests vertical transmission of endosymbionts from adult worms to offspring via the eggs. We detected a strong 16S rRNA signal lining the tegument and gastrodermis of male (Figure 1*G* and 1*H*, and Supplementary Figure 2) and female worms (Figure 1*I* and 1*J*, and Supplementary Figure 2), consistent with a recent study of adult *S. japonicum* [4]. Similar to Gobert et al [3], we did not detect a 16S rRNA signal in the ovary and vitellaria of adult females, or testes of male *S. mansoni* (Supplementary Figure 2). Nevertheless, the observation of a strong 16S rRNA signal in mature eggs, coupled with the lack of signal in immature eggs and underdeveloped embryos, points toward a technical, rather than biological, explanation. The small diameter of *Schistosoma* egg-shell pores [11] makes acquisition of bacterial cells from the external environment highly unlikely; thus, we hypothesize that schistosome reproductive tissues may harbour a small bacterial mass below the sensitivity threshold of our FISH assay. Future studies may address this hypothesis by, for example, monitoring bacterial 16S rRNA signal in developing adult reproductive tissues and throughout maturation of eggs collected from antibiotic-treated females.

**Figure 2.**
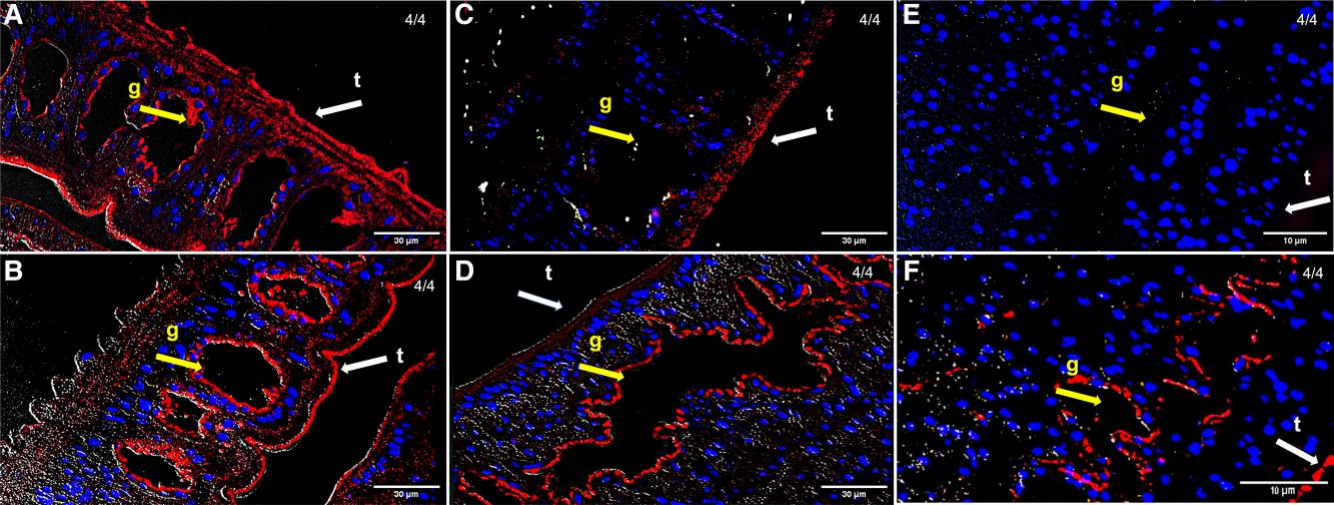
Bacterial fluorescent signal reduction in antibiotic-treated parasites. Bacterial 16S rRNA fluorescent signal (red) in representative specimens of adult *Schistosoma mansoni* at 24, 48, and 72 hours after antibiotic treatment (*A*, *C,* and *E*, respectively) and corresponding controls (*B*, *D,* and *F*, respectively). Blue, DAPI-stained nuclei. For each time point post-antibiotic treatment, the number of parasites displaying a reduced fluorescent signal, out of a total number of screened specimens, is indicated. Yellow arrow, g, gut; white arrow, t, tegument. Scale bars as indicated.

To investigate the effect of antimicrobials on the *S. mansoni*-associated microbiota, we collected and cultured mixed-sex adult worms for 72 hours in the presence or absence of an ad hoc cocktail of broad-spectrum antibiotics. No overt effect on parasite survival or fitness in worms post-incubation was observed (Supplementary Figure 3), and no 16S rRNA signal was detected in worms exposed to antibiotics for 48 and 72 hours compared to controls (Figure 2). The marked reduction and subsequent elimination of fluorescent signal in antibiotic-treated adult worms further strengthens our hypothesis of the occurrence of *S. mansoni*-associated bacteria. Associations between adult schistosomes and both gram-positive and gram-negative bacteria have been described [12]. For instance, *Salmonella paratyphi A* was demonstrated to colonize the tegument of adult *S. mansoni* recovered from the bloodstream of a human patient [13]. A later study [14] showed that the interactions between adult schistosomes and *Salmonella* occur via adherence of bacterial pili to the worm tegument, mediated by mannose-like receptors. The authors further hypothesized that adult schistosomes allow multiplication of *Salmonella* in the mesenterial system, thus accounting for the protracted course of typhoid fever in coinfected patients [14]. Interestingly, experimental infection of adult *S. mansoni* with different groups of bacteria administered intravenously resulted in varying degrees of antischistosomal activity. Selected Enterobacteriaceae (eg, *Escherichia*, *Salmonella,* and *Klebsiella*) rapidly colonized and multiplied within the schistosome coecum, resulting in worm death. Other genera (eg, *Achromobacter* and *Flavobacterium*) led to a limited colonization and temporary decrease of egg output [15]. The mechanisms by which some bacteria killed adult *S. mansoni* have not yet been established. Nevertheless, non-exclusive hypotheses include (1) parasite muscle disruption; (2) mechanical tissue damage by large numbers of bacteria colonizing the coecum; (3) secretion of bacterial endotoxin; and (4) competition for essential nutrients and/or metabolites. The latter hypothesis implies that externally acquired bacteria may compete for space and nutrients with schistosome-associated bacteria, thus possibly leading to significant disturbances in the parasite microbial homeostasis.

## CONCLUSIONS

We report the occurrence of bacteria associated with different developmental stages of *S. mansoni*. Our study sets the stage for functional characterization of these microbial communities using metagenomics approaches. However, capturing the structure of small bacterial communities colonizing helminth tissues is challenging, mainly due to the underrepresentation of target bacteria, compared with worm cells and contaminating environmental bacteria. Our initial efforts to characterize bacteria colonizing *S. mansoni* using metagenomics resulted in overrepresentation of contaminant sequences, which prevented us from drawing robust conclusions. Pre-extraction and/or post-extraction methods to enrich for microbial DNA prior to library construction, and spike-in standards added to *S. mansoni* specimens, may assist with overcoming these challenges. Insights into the structure and function of the schistosome-associated microbiota may ultimately lead to the discovery and development of novel strategies for parasite control.

## Supplementary Data

Supplementary materials are available at *The Journal of Infectious Diseases* online. Consisting of data provided by the authors to benefit the reader, the posted materials are not copyedited and are the sole responsibility of the authors, so questions or comments should be addressed to the corresponding author.

## Supplementary Material

jiad288_Supplementary_Data

## Notes

***Author contributions.*** F. F., A. C., G. R., and C. C. conceived the study and obtained funding. F. F., M. D., and G. R. performed the experiments and analyzed the data. F. F., G. R., and C. C. wrote the first draft. A. C., S. S., J. P., and M. B. provided comments to the draft. All authors read and approved the final draft.

***Acknowledgments*.** The authors express their gratitude to Simon Clare, Catherine McCarthy, Katherine Harcourt, Cordelia Brandt, and Charlotte Tolley for technical assistance with rodent experimental infections and collection of parasite specimens.

***Disclaimer.*** The funders did not participate in the design and conduct of the study, in the collection, management, analysis, and interpretation of the data, in the preparation, review or approval of the final draft, or in the decision to submit the manuscript for publication.

***Financial support*.** This work was supported by the IRCCS Sacro Cuore Don Calabria, Verona Italy (MPhil scholarship to F. F.); UK Research and Innovation (UKRI; Future Leaders Fellowship to G. R.); and the Ministry of Health Italy Fondi Ricerca Corrente-L3, P2 to IRCCS Sacro Cuore Don Calabria Hospital, Verona, Italy. C. C.’s laboratory is supported by the UKRI and the Isaac Newton Trust. Funding to pay the Open Access publication charges for this article was provided by the University of Cambridge.

***Potential conflicts of interest*.** All authors: No reported conflicts of interest. All authors have submitted the ICMJE Form for Disclosure of Potential Conflicts of Interest. All authors have submitted the ICMJE Form for Disclosure of Potential Conflicts of Interest. Conflicts that the editors consider relevant to the content of the manuscript have been disclosed.
