# Supplementary figures and images for: The Human Blood Fluke, Schistosoma mansoni, Harbors Bacteria Throughout the Parasite's Life Cycle
Source: J Infect Dis. 2023 Jul 24;228(9):1299–303. doi: 10.1093/infdis/jiad288 (PMC10629713; doi:10.1093/infdis/jiad288)

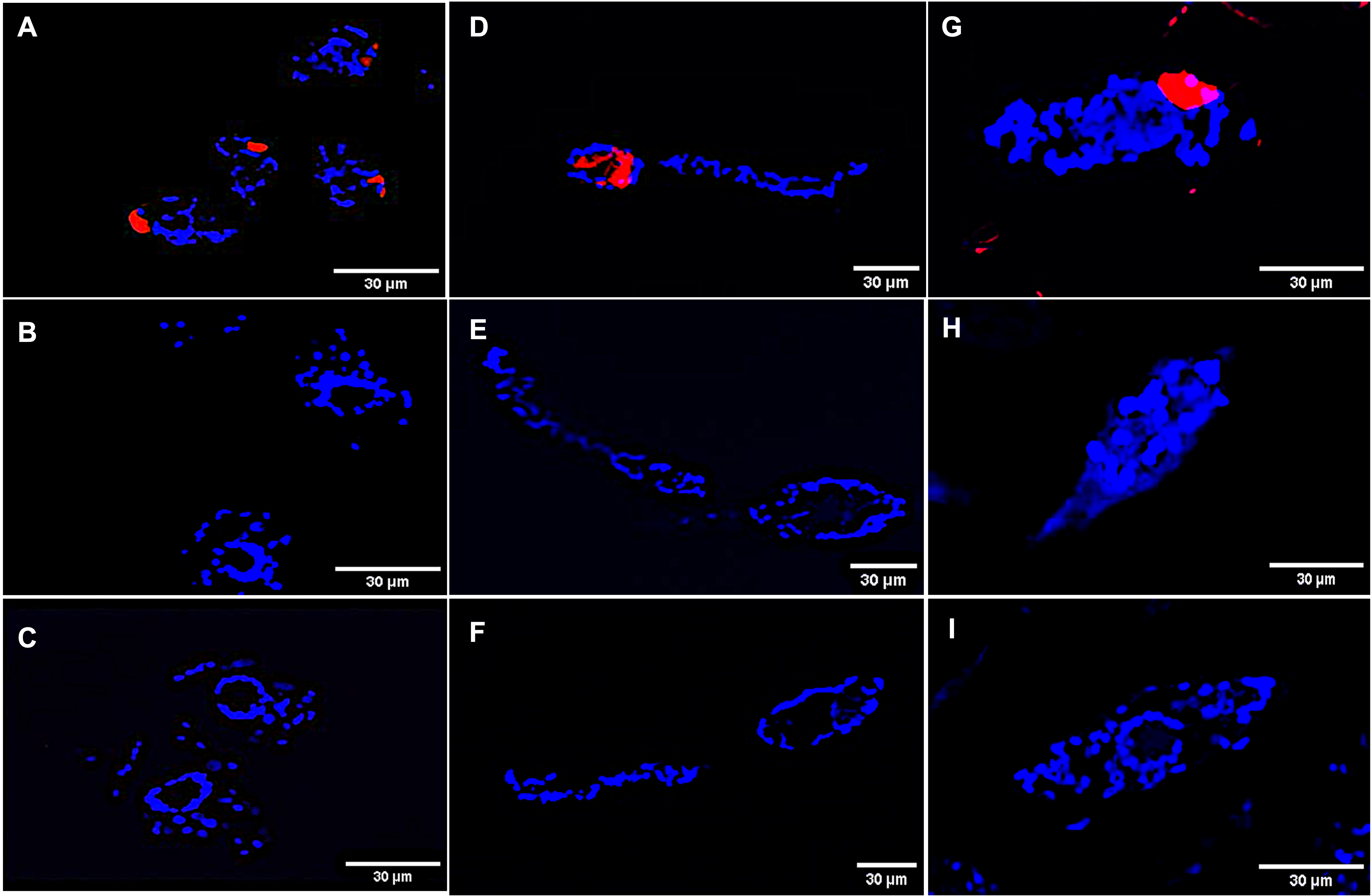

Supplement: jiad288_Supplementary_Data [file jiad288_supplementary_data.zip › Figure S1.png]

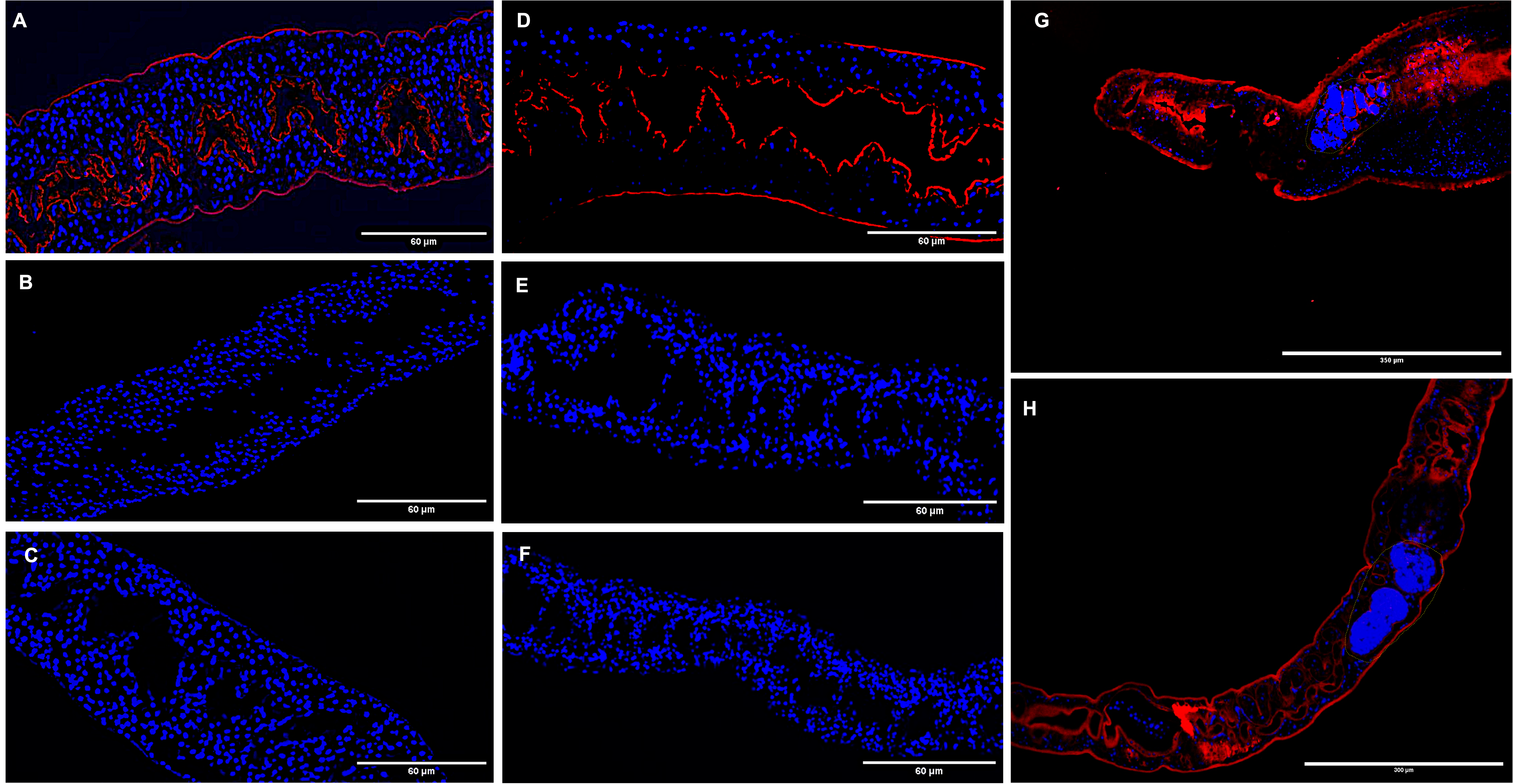

Supplement: jiad288_Supplementary_Data [file jiad288_supplementary_data.zip › Figure S2.png]

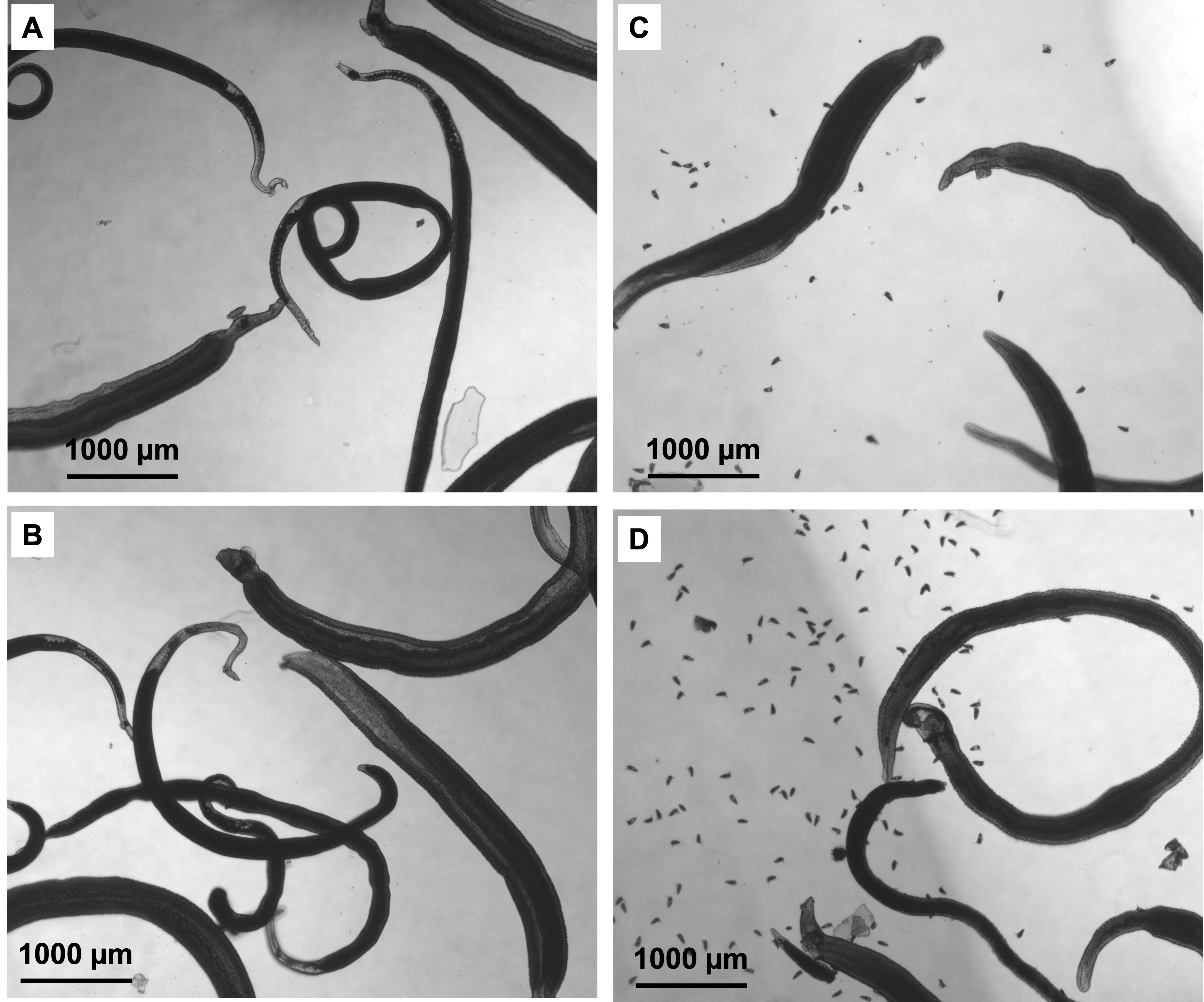

Supplement: jiad288_Supplementary_Data [file jiad288_supplementary_data.zip › Figure S3.png]
